# Effects of zinc supplementation from organic and inorganic sources on growth, blood biochemical indices, and intestinal microarchitecture in broilers

**DOI:** 10.1080/01652176.2023.2298491

**Published:** 2024-01-31

**Authors:** Muhammad Ishtiaq Khan, Naila Chand, Shabana Naz, Rasha Alonaizan, Hong Hu, Shamsuddin Shamsi, Rifat Ullah Khan

**Affiliations:** a Department of Poultry Science, Faculty of Animal Husbandry and Veterinary Sciences, The University of Agriculture, Peshawar, Pakistan; b Department of Zoology, Government College University, Faisalabad, Pakistan; c Department of Zoology, College of Science, King Saud University, Riyadh, Saudi Arabia; d College of Animal Science, Anhui Science and Technology University, Chuzhou, China; e College of Veterinary Sciences, Faculty of Animal Husbandry and Veterinary Sciences, The University of Agriculture, Peshawar, Pakistan

**Keywords:** Broilers, source of zinc, bioavailability, growth, gut health

## Abstract

In poultry nutrition, zinc supplementation is typically achieved through the addition of zinc oxide or zinc sulfate to the feed. The alternative approach of organic sources utilizes an organic ligand to bind zinc (Zn), resulting in higher bioavailability. Thus, a study was conducted to assess and compare the impact of a methionine-complexed Zn versus an inorganic Zn on growth, blood biochemical profile, gut histomorphology, and fecal excretion of Zn in broilers. The experimental design included two treatments: the addition of a zinc amino acid complex or zinc oxide to the basal diet. The zinc amino acid complex was supplemented at a dose equivalent to the inorganic zinc (Zn-80), while the organic zinc was provided at levels of 20, 40, and 80 mg/kg to a total of 400 broilers. There were five treatments in total, and each treatment was replicated four times. Broilers supplemented with an organic form of Zn at the level of 80 mg/kg had significantly (*p* < 0.05) higher body weight gain and lower feed conversion ratio (F/G). Significantly (*p* < 0.05) higher Zn excretion was recorded in broilers supplemented with inorganic Zn supplementation. Significantly (*p* < 0.05) higher villus length and width, their ratio, and lower (*p* < 0.05) crypt depth were observed in birds supplemented with 80 mg/kg organic Zn. From the results of the present study, it was concluded that Zn from an organic source at the rate of 80 mg/kg was superior in terms of growth performance, intestinal histomorphology and less excretion of Zn to the environment in broilers.

## Introduction

Zinc (Zn), a trace element, holds great importance for the survival of broilers (Khan et al. 2023). In the context of poultry nutrition, Zn plays a fundamental role in augmenting the absorption of nutrients, providing antioxidant defense, and maintaining healthy endocrine function in broilers (Naz et al. 2016; Saeeda et al. 2023). As one of the most abundant minerals in the chicken, Zn is necessary for the regulation of numerous physiological processes that impact growth, well-being and reproduction, as well as the release of hormones involved in cell division and the expression of genes responsible for protein production in broilers (De Grande et al. 2020; Besong et al. 2023). Furthermore, Zn plays a vital role in various biological processes, such as the antioxidant defense system, as well as immunological responses, among others, in broilers (Chand et al. 2020, 2021; Shao et al. 2023).

Based on performance criteria, the National Research Council (NRC, 1994) suggests that Zn should be included at a concentration of 40 mg/kg in broiler chicken diets. However, it should be noted that the NRC recommendations for minerals are primarily based on older data in broiler strains and may not cope with the requirements of modern broiler strains (Leeson and Summers, 2005). In practice, inorganic forms of minerals, including Zn, such as oxides and sulfates, are often provided to broilers at levels exceeding the NRC recommendations to optimize growth. As a result, Zn supplementation in poultry feed is commonly conducted at levels ranging from 120-180 mg/kg (Batal et al. 2001). However, it has been noted that the bioavailability of Zn from inorganic compounds is low in broilers (Zhao et al. 2014). One way to meet the Zn requirement for poultry growth can be met by adding extra amounts of Zn from inorganic sources; this can lead to high levels of Zn accumulation in broiler litter, which has detrimental environmental effects (Leeson and Caston, 2008). Moreover, higher doses of Zn supplementation are necessary for better production, and Zn excretion is increased under stressful conditions in the excreta of broilers (Sahin et al. 2009; Zhang et al. 2018). Additionally, long-term administration of Zn may result in its residues in the broiler, which can interfere with the balance of other minerals and potentially destabilize vitamins in broilers (Zhao et al. 2014).

Hence, there is a requirement for Zn sources with high bioavailability. This can potentially lead to a reduction in the supplementary Zn dosage for broilers (Kumar et al. 2021). To avoid the risk of providing excessive dietary Zn to non-ruminants while ensuring their health and performance, dietary strategies should be implemented for poultry and pigs (Ferket et al. 2002). One such strategy is the replacement of inorganic Zn with chelated sources of Zn, which can help prevent excessive Zn supplementation. In organic sources of Zn, it is bound to an organic ligand, commonly a peptide, amino acid, or protein (Chand et al. 2020). These organic forms of Zn exhibit greater bioavailability compared to inorganic Zn sources. When compared to their corresponding inorganic mineral sources, amino acid chelates exhibit higher physical heterogeneity and chemical stability. These organic forms have more efficient absorption within the gut because of diminished interactions with other components present in the ratio of laying hens (Stefanello et al. 2014). De Grande et al. (2020) reported that amino acid-based organic Zn at 60 ppm improved growth and villus length in broilers compared to Zn-sulphate. Dong et al. (2023) found that methionine hydroxyl analogue chelated zinc at the level of 90 mg/kg improved production performance in broilers fed with a low-protein diet. Chand et al. (2020) concluded that Zn-methionine at the doses of 50 and 60 mg/kg enhanced performance, intestinal histology, and paraoxonase activity in broilers compared to Zn-sulphate. Zhu et al. reported that organic Zn at the rate of 60 mg/kg improved growth performance in broilers compared to Zn-sulphate. Therefore, there is conflicting evidence for adding organic Zn to the diet of broilers. Furthermore, in the majority of prior studies, Zn sulfate has been employed, whereas limited information is available regarding the utilization of ZnO in the diet of broiler chickens. In this context, the inclusion of organic Zn in the diet has the potential to yield significant advantages in terms of achieving higher performance levels and reducing environmental concerns associated with Zn excretion. The effectiveness of different organic and inorganic Zn sources in improving broiler performance and health has been a subject of debate because of conflicting data in the literature. The aim of this study was to compare inorganic Zn (zinc oxide) with different levels of organic Zn on production performance, fecal Zn excretion, blood biochemical indices, and intestinal histology in broilers.

## Materials and methods

### Experimental design and experimental treatment

A total of 400 male Ross 308 broilers at one day of age were randomly distributed across 10 floor pens and accommodating 40 broilers per pen. The broilers were housed in floor pens with a solid flooring surface that was covered with wood shavings at a rate of 2.50 kg/m^2^. During the initial 7 days, the broilers were exposed to a light regimen of 23 h of light followed by 1 h of darkness. Starting from day 7, the broilers were subjected to a modified light schedule consisting of 18 h of light followed by 6 h of darkness. The basal diet, adhering to the recommendations set forth by the NRC (1994), contained 40 mg of Zn/kg of feed (Table 1). This diet served as the control group. The remaining four groups received supplementation of either inorganic Zn (40 mg/kg) or organic Zn (in the form of Zn-methionine; MINTREX®Zn) added to the basal diet. The graded doses of organic Zn supplementation were 20, 40, and 80 mg/kg, respectively. As a result, the treatments on the basis of final concentrations of Zn in the diets were as follows:

**Table 1.** Dietary composition of the diets.

	Starter diet (1–21 days	Finisher diet (22–35 days)
*Ingredient (%)*		
Maize	54.6	59.62
Soybean meal (48)	29	24.12
Soybeans	7.97	7.6
Rapeseed meal	2.1	2.1
Animal fat	2.5	2.5
Soy oil	1.2	1
(standared level)^1^	1.0	1.0
CaCO3	0.78	0.901
Di-Ca-phosphate	0.63	0.354
NaCl	0.25	0.22
Na-bicarbonate	0.102	0.151
L-Lys-HCl (99%)	0.16	0.175
DL-Methonine	0.256	0.208
L-Threonine	0.071	0.064
Phytase	0.02	0.02
*Calculated nutrient analyzed*		
Crude protein (%)	22.90	20.95
Crude fat (%)	6.51	6.32
Non-soluble polysaccharides (%)	15.12	15.51
Metabolizable energy (MCal/kg)	2.59	2.64
Dig. Lysine (%)	1.11	1.02
Dig. Methionine + Cysteine (%)	0.84	0.78
Dig. Threonine (%)	0.72	0.65
Dig. Valine (%)	0.90	0.82
Ca (%)	0.84	0.81
Available P (%)	0.41	0.34
NaCl + KCl (mEq/kg)	253	223
Linoleic acid (18:2) (%)	2.11	2.06

^1^ Provided per kilogram of the complete diet: vitamin D3, 2400 IU; vitamin A (from vitamin A acetate), 12,00 IU; vitamin B1, 2.5 mg; vitamin K3, 2.5 mg; vitamin B6, 3.5 mg; vitamin B2, 5.5 mg; vitamin B12, 20 µg; vitamin E (from DL-a-toco-pheryl acetate), 25 IU; folic acid, 1.5 mg; calcium pantothenate, 9 mg; niacin, 26 mg; biotin, 55 µg; Cu (as CuSO4 H2O), Fe (as FeSO4 7H2O), 45 mg; Mn (asMnSO4 H2O) 100 mg, Zn (ZnO), 60 mg; Mg (MgO) as 1600 mg.

1. Control (40 mg inorganic Zn)
2. ZnO-80 (80 mg inorganic Zn in the form of zinc oxide)
3. ZnM-20 (Zn-methionine, 20 mg/kg)
4. ZnM-40 (Zn-methionine, 40 mg/kg)
5. ZnM-80 (Zn-methionine, 80 mg/kg)

The starter diet, in the form of crumbles, was administered to the subjects starting from day 0 until day 14. Following this, the finisher diets were provided in pellet form from day 15 until day 35. Both feed and drinking water were made available to the subjects in unrestricted quantities, allowing them to consume ad libitum. On a weekly basis, the body weight gain, feed intake, and feed conversion ratio (F/G) of each pen were determined by weighing all broilers and any remaining feed. Mortality was counted as occurred.

### Zinc excretion

At 35 days of age, litter samples were collected from randomly selected chicks belonging to all treatment groups. The collected litter samples were subsequently dried in an oven at 55 °C for 72 h. Following the drying process, the samples were ground using stainless steel mills. The determination of Zn content in the samples was carried out using an Inductively Coupled Plasma Atomic Emission Spectrometer (ICP-AES) after digestion in concentrated HNO_3_.

### Blood biochemical analysis

At the conclusion of the experiment, five birds were randomly chosen from each replicate for the purpose of collecting blood samples. Serum was obtained from the blood samples through centrifugation at 3000 rpm for a duration of 10 min. Subsequently, the serum samples were stored at refrigerated temperatures and reserved for subsequent analyses of Malondialdehyde (MDA) levels and antibody titers. The determination of MDA levels in the serum samples was conducted utilizing the protocol established by Safiullah et al. Furthermore, the measurement of antibody titers against Newcastle disease (ND) was accomplished using the methodology elucidated by Ahmad et al. , using hemagglutination inhibition assay (HIA). Serum total protein, glucose, and alanine aminotransferase (ALT) were quantified using a chemistry analyzer (IRMECO Model, U 2020, Germany).

### Intestinal histomorphology

At the conclusion of the study (35 days), three broilers from each pen were euthanized using humane methods. Tissue samples were collected from the ilium and fixed in a 4% formaldehyde solution and then sectioned into 4 μm slides for subsequent hematoxylin-eosin staining. The resulting sections were examined using a light microscope. To assess villus morphology, villus length, villus width, crypt depth, and their ratio were measured on 10 randomly selected villi per sample using an automatic image analyzer.

### Statistical analysis

The data were subjected to statistical analysis using a one-way analysis of variance (ANOVA) procedure in SPSS version 20.0. The independent variable in the analysis was the dietary treatment. The results are presented as means ± standard error of the mean (SEM). To identify significant differences between treatments, a post-hoc Tukey test was performed. The significance level was set at *p* < 0.05, indicating statistical significance.

## Results

As evident from Table 2, the addition of organic and inorganic Zn to broiler feed had a nonsignificant effect on feed intake. As shown in Table 3, the body weight gains, except in the first week, were significantly impacted by the addition of zinc methionine and zinc oxide to the feed. Weight gain was increased with the increasing level of organic Zn from 20 mg onward. Group ZnM-80 gained the highest weight during the second week, followed by ZnM-40, while lower and the same BWG was recorded for groups ZnO-80, ZnM-20, and the control group. During the third, fourth, and fifth weeks, the same trend was recorded as the second week, where maximum BWG was observed for group ZnM-80, followed by group ZnM-40. The total weight gain was recorded higher for group ZnM-80, followed by ZnM-40 and ZnO-80, while the least weight gain was observed in groups ZnM-20 and the control group.

**Table 2.** Effect of organic and inorganic sources of Zn on feed intake (g/chick) in broilers.

Groups	1st **week**	2nd **week**	3rd **week**	4th **week**	5th **week**	Overall
**Control**	130 ± 1.15	360 ± 1.73	615 ± 2.30	878 ± 1.73	1182 ± 2.30	3165 ± 1.15
**ZnO-80**	133 ± 0.57	355 ± 1.73	617 ± 1.73	876 ± 1.20	1179 ± 1.85	3161 ± 1.52
**ZnM-20**	130 ± 1.73	358 ± 1.73	618 ± 3.46	875 ± 1.15	1181 ± 1.73	3162 ± 2.04
**ZnM-40**	131 ± 1.15	354 ± 2.88	620 ± 2.30	873 ± 2.02	1181 ± 3.46	3158 ± 1.85
**ZnM-80**	133 ± 0.57	360 ± 2.83	622 ± 1.73	872 ± 2.90	1175 ± 1.73	3163 ± 1.52
**P value**	0.202	0.269	0.343	0.190	0.290	0.376

ZnO, zinc oxide; 80 mg/kg feed, ZnM, zinc methionine; 20**–**80 mg/kg feed.

**Table 3.** Effect of organic and inorganic sources of Zn on weight gain (g/chick).

Groups	1st **week**	2nd **week**	3rd **week**	4th **week**	5th **week**	Total
**Control**	102 ± 0.57	231^c^ ± 2.31	391^c^ ± 1.15	506^c^ ± 2.31	618^c^ ± 2.31	1848^d^ ± 3.78
**ZnO-80**	105 ± 1.73	234^c^ ± 0.57	395^c^ ±2.30	509^c^ ± 1.73	621^c^ ± 1.73	1864^c^ ± 2.30
**ZnM-20**	102 ± 1.15	232^c^ ± 1.15	393^c^ ± 2.30	508^c^ ± 1.15	619^c^ ± 1.73	1854^d^ ± 2.64
**ZnM-40**	104 ± 0.57	241^b^ ± 1.45	401^b^ ± 1.15	515^b^ ± 1.73	628^b^ ± 1.15	1890^b^ ± 2.51
**ZnM-80**	103 ± 1.15	253^a^ ± 1.73	420^a^ ± 1.16	537^a^ ± 2.30	636^a^ ± 1.73	1949^a^ ± 2.00
**P-value**	0.3204	0.000	0.000	0.000	0.000	0.000

Groups: Control, ZnO-80 = 80 mg ZnO/kg feed; ZnM-20 = 20 mg ZnM/kg feed; ZnM-40 = 40 mg ZnM/kg feed; ZnM-80 = 80mg/kg feed. Values in the same column having no same superscript are different statistically.

^a-d^Values in the same column having no same superscript are different statistically.

Supplementation of ZnO and ZnMet affected overall and weakly F/G significantly (*p* < 0.05), as shown in Table 4. The feed conversion ratio (F/G) was improved with the increasing level of organic Zn. During the 2nd week, the F/G of group ZnM-80 and ZnM-40 improved significantly (*p* < 0.05) in comparison to ZnO-80, ZnM-20, and the control group. In the 3rd week, the maximum and same F/G was recorded for the control group and ZnM-20 followed by ZnO-80 and ZnM-40, while group ZnM-80 had the minimum F/G. In the 4th week, the F/G of group ZnM-80 and ZnM-40 differed significantly (*p* < 0.05) from ZnO-80, ZnM-20, and the control. In the 5th week, ZnM-80 and ZnM-40 had significantly (*p* < 0.05) lower (good) F/G as compared to ZnO-80, ZnM-20, and control. Overall, F/G was maximum (worst) and the same for the control and group ZnM-20, followed by ZnO-80 and ZnM-40.

**Table 4.** Effect of organic and inorganic sources of Zn on feed conversion ratio of broilers.

Groups	1st **week**	2nd **week**	3rd **week**	4th **week**	5th **week**	Overall
**Control**	1.27 ± 0.01	1.55^a^ ± 0.12	1.57^a^ ± 0.01	1.73^a^ ± 0.04	1.91^a^ ± 0.08	1.70^a^ ± 0.01
**ZnO-80**	1.26 ± 0.01	1.51^b^ ± 0.05	1.55^ab^ ± 0.00	1.71^a^ ± 0.01	1.89^ab^ ± 0.02	1.69^b^ ± 000
**ZnM-20**	1.27 ± 0.01	1.54^ab^ ± 0.08	1.57^a^ ± 0.03	1.72^a^ ± 0.11	1.90^a^ ± 0.06	1.70^a^ ± 0.03
**ZnM-40**	1.25 ± 0.03	1.46^c^ ± 0.02	1.54^b^ ± 0.01	1.69^b^ ± 0.08	1.87^b^ ± 0.10	1.66^c^ ± 0.02
**ZnM-80**	1.28 ± 0.02	1.42^d^ ± 0.03	1.48^c^ ± 0.00	1.62^c^ ± 0.10	1.84^c^ ± 0.00	1.62^d^ ± 0.08
**P value**	0.684	0.000	0.000	0.000	0.001	0.000

Groups: Control, ZnO-80 = 80 mg ZnO/kg feed; ZnM-20 = 20 mg ZnM/kg feed; ZnM-40 = 40 mg ZnM/kg feed; ZnM-80 = 80mg/kg feed. Values in the same column having no same superscript are different statistically.

As shown in Table 5, Zn content in the excreta of the broilers increased significantly (*p* < 0.05) with Zn doses. The concentration of Zn per kg of excreta of chicks offered Zn from organic sources was significantly (*p* < 0.05) lower than that of birds fed with Zn from inorganic sources, as evident from group ZnM-80 as compared to group ZnO-80. The highest amount of Zn was observed in the excreta of ZnO-80 followed by ZnM-80, ZnM-40, and ZnM-20, while the control group excreted the lowest amount of Zn in excreta. No significant difference was found in blood MDA, antibody titer, serum glucose, total protein, and alanine aminotransferase in the control and experimental groups of birds. There was no significant difference in the mortality of birds in the control and supplemented birds.

**Table 5.** Effect of organic and inorganic zinc on excreta zinc, blood biochemical profile, antibody titer, and melanodialdehyde of broilers fed organic or inorganic zinc.

Groups	Zinc content of excreta (mg/kg)	Alanine aminotransferase (U/L)	Serum glucose (mg/dL)	Total proteins (g/dL)	Antibody titer against ND	MDA (nmol/ml)	Mortality %
Control	202^e^ ± 1.73	3.61	285	3.15	5.87 ± 0.01	2.78 ± 0.19	1.9 ± 0.21
ZnO-80	270^a^ ± 2.88	3.66	290	3.21	5.91 ± 0.02	2.85 ± 0.21	1.85 ± 0.12
ZnM-20	218^d^ ± 0.57	3.59	292	3.18	5.96 ± 0.01	2.76 ± 033	1.83 ± 0.18
ZnM-40	235^c^ ± 1.15	3.62	291	3.17	5.99 ± 0.03	2.77 ± 0.11	1.80 ± 0.17
ZnM-80	257^b^ ± 2.30	3.63	289	3.15	6.01 ± 0.04	2.76 ± 0.12	1.89 ± 0.2
P value	0.0000	0.12	0.06	0.17	0.91	0.88	1.88 ± 0.15

Groups: Control, ZnO-80 = 80 mg ZnO/kg feed; ZnM-20 = 20 mg ZnM/kg feed; ZnM-40 = 40 mg ZnM/kg feed; ZnM-80 = 80mg/kg feed. Values in the same column having no same superscript are different statistically.

**Figure 1.**
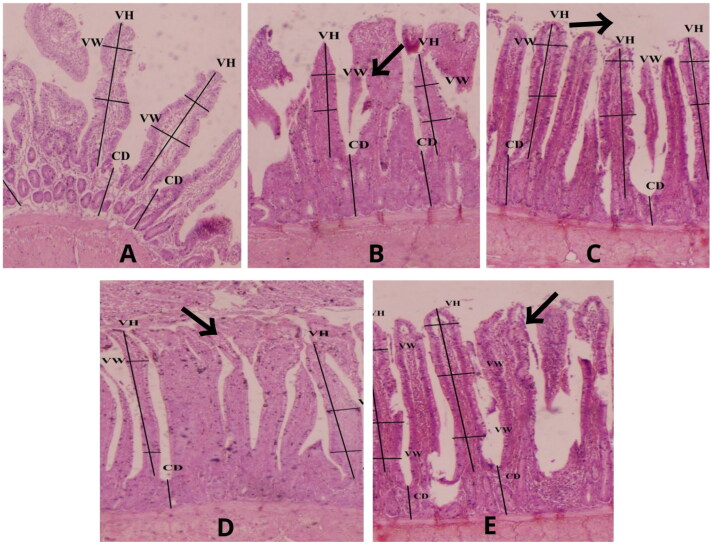
Histological microphotograph of ilium of the control (a), ZnO (B), ZnM 20 (C), ZnM 40 (D), and ZnM 80 (E) of broilers.

Table 6 shows the histomorphology of the ileum of birds having organic and inorganic Zn in the feed. The VH showed a significantly (*p* < 0.05) high value with the higher supplementation rates of organic Zn. The greatest villus height was seen in group ZnM-80 (Figure 1), followed by ZnM-40. There was a nonsignificant (*p* > 0.05) difference among the VH of group ZnO-80, ZnM-20, and the control group. The CD was significantly (*p* < 0.05) decreased with the supplementation of higher levels of organic sources of Zn. Group ZnO-80, ZnM-20, and the control group showed significantly (*p* < 0.05) the same CD. The VW was greatest in group ZnM-80, followed by ZnM-40. The ratio of VH and CD was higher in group ZnM-80, followed by ZnM-40. Statistically, the same VH to CD ratio was reported for group ZnO-80, ZnM-20, and the control group.

**Table 6.** Intestinal histomorphology of broilers fed with organic and inorganic zinc in the ration.

Groups	Villus height (µm)	Villus width (µm)	Crypt depth (µm)	VH:CD
**Control**	701^c^ ± 1.15	136^c^ ± 0.57	224^ab^ ± 0.57	3.12^d^ ± 0.03
**ZnO-80**	705^c^ ± 2.31	140^c^ ± 1.54	221^b^ ± 1.15	3.18^c^ ± 0.01
**ZnM-20**	702^c^ ± 0.57	139^c^ ± 1.54	225^a^ ± 0.57	3.11^d^ ± 0.01
**ZnM-40**	830^b^ ± 1.73	146^b^ ± 1.73	217^c^ ± 1.15	3.82^b^ ± 0.04
**ZnM-80**	916^a^ ± 1.15	151^a^ ± 1.73	210^d^ ± 1.15	4.36^a^ ± 0.03
***p* value**	0.0000	0.0001	0.0063	0.0000

Groups: Control, ZnO-80 = 80 mg ZnO/kg feed; ZnM-20 = 20 mg ZnM/kg feed; ZnM-40 = 40 mg ZnM/kg feed; ZnM-80 = 80mg/kg feed. Values in the same column having no same superscript are different statistically.

## Discussion

In this study, feed intake in broilers was not significantly affected by the supplementation of organic and inorganic forms of Zn in the control and experimental groups. However, body weight gain and F/G were significantly affected by the two sources. Resultantly, the highest weight body gain and F/G were reported in ZnM-80. It seems that the increase in these parameters was not due to feed intake but higher body weight with an organic source of Zn. It was evident that the organic form of Zn demonstrated superior effectiveness in comparison to the inorganic form. Furthermore, a dose-dependent response was observed for both forms of Zn. The organic Zn perform a chelating role, resulting in enhanced absorption and utilization of nutrients from feed within the body while also reducing the excretion of essential nutrients (Yusof et al. 2019; Chand et al. 2020). The enhanced effect observed through organic Zn supplementation can be attributed to its elevated bioavailability, leading to enhanced nutrient digestibility. The improved performance in birds treated with Zn has also been linked to its positive influence on the secretion of digestive enzymes (Naz et al. 2016; Ogbuewu and Mbajiorgu, 2023). Similar to our study, De Grande et al. (2020) found that organic zinc derived from amino acids, administered at a concentration of 60 ppm, led to enhanced growth in broiler chickens compared to the use of zinc sulfate. In a separate study, Dong et al. (2023) observed that utilizing methionine hydroxyl analog chelated Zn at a dosage of 90 mg/kg improved production performance in broilers that were fed a low-protein diet. Additionally, Chand et al. (2020) reached the conclusion that supplementing the diet of broilers with Zn-methionine, administered at dosages of 50 and 60 mg/kg, resulted in improved performance compared to the utilization of Zn-sulfate. Furthermore, Zhu et al. reported that administering organic Zn at a rate of 60 mg/kg led to enhanced growth performance in broilers in comparison to the use of zinc sulfate.

Additionally, the organic form of Zn is considered more environmentally friendly. Broilers fed a diet supplemented with organic Zn exhibited lower fecal excretion of Zn compared to those fed an inorganic Zn. This can be linked to the improved bioavailability of the organic form of Zn, leading to enhanced absorption and utilization within the body. The excreted zinc content was notably elevated in birds that were provided with the basal diet supplemented with 80 mg/kg of Zn compared to the control group. These findings align with prior research that suggests higher dietary Zn levels are associated with increased Zn quantities in excreted matter (Mwangi et al. 2017; Zhang et al. 2018; De Grande et al. 2020). The Zn excretion observed in this study highlights that the inorganic form of Zn is more predisposed to biological excretion, whereas the organic form of Zn demonstrates greater retention and reduced excretion. The minimal Zn excretion stemming from organic sources serves as a positive indicator of reduced environmental pollution.

Serum biochemistry parameters serve as crucial standards of either physiological or pathological alternations, nutritional status, and total health status in broilers. Zinc plays a vital role in insulin synthesis, secretion, and storage, as well as in regulating glucose levels in the blood (Søndergaard et al. 2006). This finding aligns with the study of Zakaria et al. (2017), which reported that Zn supplementation did not influence serum glucose and total protein. Likewise, our results are consistent with the findings of Lu and Combs (1988), who observed that Zn supplementation (ZnO) in broiler chicks did not impact glucose levels. Furthermore, Kim and Kang (2022) highlighted that Zn has a critical role in protein metabolic wastes and degradation and concluded that Zn did not influence serum glucose, total protein, and ALT levels in broiler chickens. However, in our study, no significant difference was observed in serum glucose, total protein, and ALT levels. The MDA has been identified as an ideal marker of oxidative stress induced by free radicals. De Grande et al. (2020) found no differences in plasma MDA levels at slaughter age (day 36) in broilers. Zinc is recognized for its ability to activate antibody production and assist in clearing foreign substances. In its organic form, zinc (Zn) has also been linked to enhancing IgA and IgG, as reported by Jarosz et al. (2017). Moreover, the same study by Jarosz et al. (2017) highlights that Zn-Gly, when compared with the inorganic form of zinc in broilers, contributes to a more potent immune system through the generation of inflammatory molecules and immunoglobulins. In addition, Chand et al. (2020) also reported that organic Zn resulted in better immune response against ND. These results do not agree with our findings. The reason could be that the dose, duration, genetic makeup, and other experimental conditions of the experiment may have a role in influencing these parameters.

In a similar manner, the type of Zn supplementation significantly influenced the villus dimensions in the ileum in the current experiment. The organic form of Zn demonstrated superior effects on the histology of the ileum compared to the inorganic form. Previous studies conducted by various research groups have also reported improved villus dimensions in broilers when supplemented with both sources of Zn (Chand et al. 2020; De Grande et al. 2020). In this study, the enhanced measurements of the intestinal microstructures may be associated with greater surface area for absorption and a higher proliferation rate of crypt cells within the villi. This effect is likely a result of the Zn supplementation, as suggested by Chand et al. (2020). The crypts within the intestinal lining undergo a continuous process of cell renewal, wherein new cells migrate from the crypts towards the tip of the villi. Throughout this migration, the cells mature and enhance their ability to efficiently absorb nutrients. (De Grande et al. 2020). An increase in the length of the villi is linked with better digestion and absorption of nutrients brought by higher brush border nutrient transport systems. This relationship was highlighted by Collet (2012), who stated that the efficiency of digestion is directly correlated with the surface area of the intestines, absorption, and, ultimately, feed conversion. Considering this, the observed improvement in villus morphology, particularly when supplementing with organic Zn complexes, may partially account for the lower F/G during the starter and grower phases.

## Conclusion

It is evident from these results that an organic source of Zn at the rate of 80 mg/kg was superior in improving growth and intestinal histomorphology in broilers.

## Data Availability

On request.
